# Anxiety, Working Memory, and Academic Achievement in Neurodivergent and Neurotypical Children: A Preliminary Study

**DOI:** 10.3390/children13101440

**Published:** 2026-10-21

**Authors:** Keira Goulding, Emily Freeman

**Affiliations:** Discipline of Psychological Sciences, The University of Newcastle, Callaghan, NSW 2308, Australia; keira.goulding@uon.edu.au

**Keywords:** anxiety, academic achievement, reading comprehension, mathematics, working memory, neurodivergent children, autism, ADHD

## Abstract

**Highlights:**

**What are the main findings?**

- Neurodivergent children had higher parent-reported ASC-ASD scores than their neurotypical peers. No statistically significant group differences were detected in working memory accuracy or most academic achievement outcomes. However, exploratory subgroup analyses suggested that autistic participants had lower average Reading Comprehension scores.
- Preliminary analyses did not detect statistically significant associations between anxiety and either working memory or academic achievement in reading or mathematics, although working memory was related to academic achievement.

**What are the implications of the main findings?**

- Findings emphasise the need for larger studies to clarify how cognitive and affective factors contribute to academic outcomes for neurodivergent students, particularly those with co-occurring neurodivergent profiles.
- Future work should explore how assessment environments and cognitive demands interact with affective factors for neurodivergent students, and whether supportive educational and assessment contexts help reduce barriers to learning and achievement.

**Abstract:**

Background/Objectives: Anxiety is highly prevalent amongst neurodivergent children, yet little research has examined how anxiety relates to academic achievement in neurodivergent students, nor how working memory, a key cognitive predictor of achievement, is concurrently associated across neurotypes. This cross-sectional study aimed to provide a preliminary investigation into the associations between anxiety, working memory and academic achievement in reading and mathematics in a sample of neurodivergent and neurotypical children. Understanding these relationships is important in both child mental health and educational contexts, as anxiety may influence how neurodivergent children engage with and perform on cognitive and achievement tasks. Methods: Forty-seven children aged 8–13 years completed the Working Memory Power Test (WMPT) and four subtests from the Wechsler Individual Achievement Test-Third Edition (WIAT-III). Parents completed the Anxiety Scale for Children with Autism Spectrum Disorder (ASC-ASD). Group differences were examined, and whole-sample correlational and regression analyses were conducted to explore the relationships between anxiety, working memory, and academic achievement. Exploratory subgroup correlations were also examined. Results: Neurodivergent children (*n* = 18) scored higher than their neurotypical peers (*n* = 29) on the ASC-ASD; however, no significant differences were observed in working memory accuracy scores. Achievement scores did not differ significantly across groups, although autistic children showed lower average scores in Reading Comprehension. Whole-sample correlational and regression analyses did not detect statistically significant associations between anxiety and either working memory or achievement, although working memory showed positive associations with Reading Comprehension, Numerical Operations and Math Problem Solving (ρ = 0.34–0.53). Exploratory subgroup correlations did not remain significant after false discovery rate correction. Conclusions: These preliminary findings suggest that ASC-ASD scores were not significantly associated with working memory or academic achievement in this sample, while working memory showed the expected links with several achievement domains. Larger studies are needed to clarify potential cognitive and affective contributors to academic outcomes for neurodivergent students, and to examine how environments and task demands may interact with affective factors to influence academic engagement and performance.

## 1. Introduction

Anxiety disorders are one of the most common mental health conditions that manifest in childhood, affecting approximately 6.5 to 8.5% of children and adolescents globally [1,2]. For neurodivergent children, there is an evident increased risk of anxiety, with meta-analyses finding that approximately 18.4% of ADHD children [3] and 39.6% of autistic children [4] have co-occurring anxiety disorders. Although the specific focus or triggers of anxiety symptoms can vary across subtypes, anxiety disorders are generally characterised by feelings of excessive worry or apprehension that are difficult to control and significantly interfere with an individual’s ability to participate in daily life [5]. Other types of anxiety, such as test anxiety [6] and school anxiety [7], are also marked by similar emotional patterns of intense worry and tension relating to specific situations or events. Regardless of the triggering stimulus, these emotions can produce physical and cognitive symptoms such as breathlessness, irritability, difficulties concentrating, muscle tension, restlessness, fatigue, and sleep difficulties [5].

Given this high prevalence, it is necessary to consider the potential negative impacts often associated with anxiety, as these can be significant and severe. For example, anxious neurotypical youth tend to report poorer quality of life [8], higher rates of school distress [9], and greater peer victimisation [10] compared to their non-anxious peers. These impacts are also not limited to pre-adult life, as individuals diagnosed with anxiety during childhood are more likely to experience future anxiety, mood and substance use disorders, as well as report poorer financial, occupational, educational and physical health outcomes in adulthood [11].

Similar findings have been observed in neurodivergent youth, with research indicating that anxiety detrimentally impacts the quality of life of autistic [12] and ADHD children [13]. For autistic children specifically, difficulties with uncertainty may be the primary driver of this relationship [14]. Peer relationships and social competence have also been inconsistently related to anxiety in autistic children, although many research designs use measures which require autistic participants to behave neurotypically to avoid the assumptions of deficits, especially for social competence [15,16]. Other research has also suggested that anxiety limits engagement in activities both at home and in the community [17], and is associated with school distress in neurodivergent children [18]. As the school environment is often one that is poorly matched to the needs of neurodivergent students, it is unsurprising that many children struggle with anxiety in this setting [19,20,21].

One area that has received substantial attention in the literature is the link between anxiety and academic achievement in neurotypical children [22,23,24]. This includes both specific forms of anxiety related to the school setting and academic performance, such as test anxiety [25,26] and math anxiety [27], as well as anxiety disorder sub-types such as separation anxiety [28], social anxiety/phobia [29,30] and generalised anxiety [31]. Although findings from individual studies sometimes fail to find a direct association, the general trend across the literature indicates that higher levels of anxiety tend to be associated with poorer academic performance in the domains of mathematics and reading, as well as an increased risk of early school termination and poorer academic self-concept [25,32,33,34].

In autistic children, academic achievement research has predominantly explored the associations with intellectual ability [35,36,37,38], sensory processing [39,40], or executive functioning (Ref. [41]; for an overview of the most recent literature relating to educational outcomes in autistic students, see Smart et al. [42]). Similarly, the achievement literature has largely focused on the role of attention and hyperactivity, or treated versus untreated outcomes for ADHD children [21,43]. Regardless of the domain assessed, the results commonly indicate that neurodivergent students perform more poorly, on average, than their neurotypical peers [43,44]. However, despite the high prevalence of anxiety within neurodivergent populations, little research has explored the relationship between anxiety and academic achievement for both autistic and ADHD children concurrently to determine whether anxiety contributes to these observed differences. Furthermore, this gap is particularly evident for autistic students.

For example, a systematic review in 2021 found only three studies that reported on both anxiety and academic outcomes for autistic children [16]. Of these studies, two observed poorer perceived school functioning in autistic children with elevated anxiety [12,14], while the third found that higher levels of test anxiety were negatively, but not significantly, associated with performance in mathematical problem-solving [45]. Few studies have since added to these findings. Of those that have been published, one paper found that while there was no significant difference for trait math anxiety between autistic and neurotypical school-aged boys, the autistic boys in the sample reported higher worries, used as a measure of state anxiety related to the assessment context, and performed more poorly in a mathematics task with time constraints [46]. The authors suggested that a stress response was intentionally induced by the timed nature of the task, which may have impacted accuracy in autistic students and potentially led to scores which may not be reflective of their actual capabilities.

In another recent study, Ambrose and colleagues [47] asked autistic primary and high school-aged students about their perceptions of the social and academic impacts of their anxiety. Across the sample, academic outcomes were rated as the most highly impacted, as students reported that when they were worried it was harder to start, concentrate on, or do their best schoolwork. Although this is not direct evidence of an association between anxiety and academic performance, it does suggest that elevated anxiety prohibits autistic children from fully engaging in learning tasks, which may have consequences for their overall attainment.

While a larger body of research has considered how anxiety relates to academic achievement in ADHD children and youth, few studies have considered how these associations are affected by co-occurring neurodivergent profiles, for example both autism and ADHD (commonly referred to as AuDHD). This gap is particularly concerning, as a large study of 9108 students observed that individuals who had multiple neurodevelopmental conditions had an increased likelihood of poorer educational outcomes across the domains of language, mathematics, arts and physical education, compared to those with singular profiles [48].

Other research with ADHD children has produced mixed findings. For example, some studies have observed that anxiety is not related to achievement in math or reading, or with homework problems, academic productivity or academic performance [49,50,51]. In contrast, others have reported poorer reading achievement in children with co-occurring anxiety and ADHD compared to non-anxious, neurotypical controls [52]. More broadly, the role of ADHD traits such as inattention have been examined, with some research highlighting the significant interactions between harm avoidance, separation anxiety and inattention, and their effects on reading fluency [53]. Other studies have also demonstrated that ADHD children with anxiety may display better performance on tasks requiring sustained and selective attention compared to ADHD children without anxiety [54]. Taken together, these findings suggest that anxiety may interact with achievement in ADHD children in ways that are not yet well understood.

Moving beyond the direct link between anxiety and academic achievement, some research has proposed that this relationship may be mediated by cognitive factors, such as concentration [55] and inattention [22]. In neurotypical groups, one factor which has often been suggested as both a direct predictor of academic performance and mediatory mechanism is working memory [56,57,58]. One of the most prominent conceptualisations of working memory is the multicomponent model of working memory [59,60], which posits that working memory is involved in both the short-term storage and manipulation of information. The model has two domain-specific sub-components, the visuospatial sketchpad, which handles visual-spatial information, and the phonological loop, responsible for verbal and auditory information. These two components are controlled by the central executive, an attentional control system, while the episodic buffer, a later addition to the model, integrates sensory information from different modalities. As working memory is involved in the active maintenance of task-relevant information during activities that require complex cognition [61], for example learning tasks and completing multi-step instructions, it follows that working memory is very relevant to academic performance and educational outcomes.

The direct association between working memory and academic achievement in school children has been well-documented in the literature in the domains of word reading, reading comprehension, spelling, mathematical reasoning, and numerical operations [62,63,64,65,66,67,68,69,70,71,72,73]. Working memory has also been linked to achievement in samples of ADHD [74,75] and autistic children [76], however some research has not observed the anticipated relationships for reading comprehension [77,78]. Other research has also suggested that the relationships between working memory and achievement may differ depending on neurotype, with stronger relationships observed between working memory and math achievement for autistic individuals in comparison to their neurotypical peers [79], as well as between children with high versus low ADHD traits [80].

Several theories have been proposed to account for the impact of emotions or affectivity on executive functioning, including working memory. For example, Attentional Control Theory [81,82] suggests that anxiety, particularly the component of ‘worry’, may divert attentional resources away from task-relevant processing, thereby reducing the cognitive capacity available for complex tasks. Within this framework, working memory has been considered as a potential pathway through which anxiety may relate to academic performance. In neurotypical samples, researchers have found evidence consistent with this idea, with some studies observing that trait anxiety was related to increased levels of worry, which in turn impacted complex working memory performance, which is consequently associated with lower academic achievement [56,57]. However, the observed mediatory effects vary across academic domain and studies. For instance, Owens et al. [56] found that verbal working memory mediated the relationship between trait anxiety and performance in mathematics, quantitative reasoning and non-verbal reasoning, but not science, English or verbal reasoning in secondary school students. Conversely, in similarly aged students, Owens et al. [57] observed that working memory acted as a mediator between anxiety and a composite measure of academic achievement that included mathematics and science, but not English test scores.

Notably, Alfonso and Lonigan [58] also observed discrepant results, finding that working memory mediated the association between trait anxiety and passage comprehension, as well as calculation, but that all effects were positive. In other words, in this study increased anxiety was positively associated with working memory, and the relationship between working memory and academic achievement was also positive. This finding may suggest that the relationships between these variables is curvilinear, where moderate levels of anxiety may enhance academic performance, but higher levels may cause significant disruptions to cognitive processes, thereby reducing performance. Despite these differences, it appears that more complex, cognitively demanding tasks tend to rely more heavily on working memory and are therefore more likely to find a mediation effect.

Of note, there is remarkably little research simultaneously investigating the relationships between anxiety, working memory and academic achievement in neurodivergent children. For autistic children specifically, one study observed that working memory was not significantly associated with performance in mathematics once other factors including perceptual reasoning, verbal ability and test anxiety were accounted for [45]. For ADHD children, a study by Gaye et al. [83] observed no mediatory mechanisms when trait anxiety was parent or child reported, although ADHD diagnostic status and working memory accounted for a total of 65% of the variance in mathematics. Given this, it is important to investigate whether associations concurrently exist between each of these factors to determine whether further examination of cognitive–affective theories in this population may be warranted.

In sum, despite the well-established links between anxiety, working memory and academic achievement in neurotypical children, a search of the literature revealed few studies exploring anxiety and academic achievement in autistic students, and we were unable to identify any studies that concurrently examined anxiety, working memory and academic achievement in domains other than mathematics (based on a targeted search of the available literature up to September 2026). Furthermore, it is yet unclear whether these relationships differ depending on child neurotype (i.e., whether neurodivergent and neurotypical children demonstrate the same strength and direction of associations). Although mediation has been proposed in neurotypical samples, the present study focused on examining direct associations rather than mediatory mechanisms due to its preliminary nature and insufficient precision to evaluate indirect effects. Given the high prevalence of anxiety among autistic and ADHD children, as well as the reported academic difficulties, it is imperative that factors which impact educational progress are explored so that effective, neuroaffirming supports can be put in place to optimise long term outcomes. Therefore, the current research aimed to address these gaps by providing a preliminary examination into the relationships between anxiety, working memory, and academic achievement in reading and mathematics in a mixed sample of neurodivergent and neurotypical children.

Based on prior research in neurotypical samples, it was anticipated that higher anxiety would be associated with poorer working memory performance, and poorer academic achievement. In contrast, it was expected that working memory would be positively associated with academic achievement, particularly for more complex tasks.

## 2. Materials and Methods

### 2.1. Design

This study was part of a larger, cross-sectional project investigating the impact of anxiety on the school experience of children and their families. This project includes additional measures which are not reported in this paper and have not yet been published elsewhere. The present research used quantitative data collected from parents via online surveys, as well as face-to-face data collected from their children. Participants were recruited from May 2024 to June 2026. This study was not preregistered.

### 2.2. Participants

Recruitment occurred via convenience sampling, where parents self-selected to commence the online survey in response to a Facebook advertisement or a physical flyer for the study. Participants were also recruited via the University of Newcastle’s SONA database, a research management system for volunteers from the general public and university staff, as well as undergraduate psychology students who can participate for partial course credit.

After removing incomplete datasets, there were 56 parents who completed the online survey and 48 children who completed face-to-face testing. As matched child and parent data was required for the analyses in this study, parent-only or child-only participants were excluded. The final sample consisted of 47 parent-child pairs. Unmatched cases occurred when parents completed the online survey but did not attend the child testing session, or in one case where a child participant completed testing without a corresponding completed parent survey.

An initial target of approximately 100 neurotypical and 100 neurodivergent children was set, based on prior studies that detected associations between the variables of interest (anxiety, working memory, and/or academic achievement) in school-aged neurotypical samples, and to enable sufficient power for subgroup analyses, assuming medium effect sizes and α = 0.05. However, recruitment difficulties meant that this target was not reached, and the present study should therefore be interpreted as a preliminary investigation.

In the final sample, child participants ranged in age from 8 to 13 years (*M* = 9.83, *SD* = 1.67), and parent participants ranged from 29 to 54 years (*M* = 40.94, *SD* = 5.42) (One parent did not report their age.). Child neurotype was based on parent-reported clinical diagnoses (e.g., autism, ADHD, dyslexia), which were not independently verified. Ethical approval for this study was granted by the Human Research Ethics Committee of the University of Newcastle, Australia (HREC-2023-0105).

### 2.3. Measures

#### 2.3.1. Demographic Characteristics

Parent participants completed an online survey which included items about their child’s age, gender, neurodivergence, co-occurring conditions, school type, attendance difficulties, and history of school exclusion. Parents were also asked about their own age, gender, education, relationship status, employment status, geographical location, and household income.

#### 2.3.2. Anxiety

Given the anticipated prevalence of neurodiversity within the sample, the Anxiety Scale for Children with Autism Spectrum Disorder—Parent Version (ASC-ASD) [84] was chosen for its sensitivity to autism-specific anxiety presentations, such as anxiety related to sensory processing and intolerance of uncertainty. The scale consists of 24 items, each answered on a 4-point scale (Never = 0, Sometimes = 1, Often = 2, Always = 3), and divided across four subscales: Anxious Arousal (AA; 6 items), Performance Anxiety (PA; 5 items), Separation Anxiety (SA; 5 items), and Uncertainty (U; 8 items). Item scores within each subscale are summed to create subscale totals, while the total score is calculated as the sum of all items. Total scores can range from 0 to 72, with higher scores indicative of increased anxiety. The suggested cut-off for significant levels of anxiety is ≥20 for the total score. Currently, there are no recommended cut-offs for subscales.

The ASC-ASD has previously demonstrated good internal consistency and test–retest reliability [84], as well as good validity in samples of autistic children [85,86,87]. It has also been used in studies with both neurotypical and neurodivergent children [18]. However, there is not currently sufficient evidence to conclude that this measure displays similar psychometric properties when used with children of other neurotypes (e.g., ADHD, neurotypical). Given this, the ≥20 threshold score and categorical below threshold vs. meets threshold classifications applied in this study are considered exploratory and should not be interpreted as equivalent to clinical diagnostic thresholds. In line with previous results, internal consistency was excellent for the total score (Cronbach’s α = 0.92), and acceptable to good for the subscales (AA α = 0.78; PA α = 0.83; SA α = 0.86; U α = 0.88).

#### 2.3.3. Academic Achievement

The Wechsler Individual Achievement Test (WIAT-III) [88] is an individual assessment test that measures achievement in individuals aged from 4 years to 50 years 11 months. Four subtests from the WIAT-III were used to examine academic achievement in reading (Word Reading and Reading Comprehension) and mathematics (Numerical Operations and Mathematical Problem Solving) in child participants. The WIAT-III Australian and New Zealand Standardised edition was used, and all standard scores were derived from the Australian normative sample.

The Word Reading subtest involves reading aloud a list of words, measuring the accuracy of single word reading. This subtest does not require semantic understanding, but relies on word recognition, decoding skills and phonological awareness. The Reading Comprehension subtest involves reading passages and responding to verbal questions based on the text, which assesses literal and inferential comprehension skills. The Numerical Operations subtest involves solving mathematical problems of increasing difficulty, including addition, subtraction, division and multiplication operations, assessing calculation skills in untimed conditions. Lastly, the Mathematical Problem Solving subtest involves listening to a verbally presented problem, examining the accompanying visual stimuli, and responding either verbally or by pointing to the answer. This test measures reasoning ability and problem-solving skills and is also untimed.

Raw scores for each subtest are converted into standard scores based on either the child’s age or grade level. For this study, grade-level norms were used to better reflect children’s actual educational experience. As some parents of neurodivergent children may delay their child’s entry to school, or children may have repeated a grade, chronological age may not always correspond with actual time spent in formal schooling.

#### 2.3.4. Working Memory

The Working Memory Power Test (WMPT; Lewis Cadman Consulting Pty Ltd., Sydney, Australia) is an online individual working memory assessment. It assesses both accuracy and confidence in performance. In comparison to many multicomponent batteries, the WMPT has the advantage of being quick to administer, fully automated, and does not require the child to respond verbally to assess their working memory. Given that many autistic and highly anxious children, such as those with situational mutism, may communicate non-verbally or have a preference for this mode of communication, this assessment may be particularly useful for screening for working memory problems within these populations without the need for the child to verbally respond to the assessor.

The WMPT has five levels of increasing difficulty: Memorise, 1-Swap, 2-Swap, 3-Swap, and 4-Swap. In the first condition, Memorise, children are presented with three simple images of animals (e.g., cat, pig, duck), numbered from left to right 1, 2 and 3, and are asked to remember the order. On the following screen, children are shown six potential answer options consisting of various combinations of the same animals and must select the previously presented sequence.

For the swap conditions, children must complete an increasing number of mental swaps. For example, in 1-Swap, children will be asked to swap 1 with 2 (e.g., cat, pig, duck becomes pig, cat, duck). Children must then select this new order on the following screen. The maximum number of mental swaps children are required to perform is four in 4-Swap (e.g., swap 1 with 2, 3 with 1, 2 with 3, 1 with 2). For each level, children are instructed to answer as quickly and carefully as they can. After each trial, children are asked to rate their confidence in their answer on a 4-point scale (ranging from ‘I guessed the answer’ to ‘I definitely got it right’).

There are five trials per level, totalling a maximum of 25 trials. Since initial publication, a discontinue rule has been implemented where children do not progress further once incorrectly answering 3 out of the 5 items within a level. Items not administered after discontinuation are scored as incorrect, reflecting the assumption that children who met this rule were unlikely to answer more difficult items correctly. Accuracy is reported as a percentage, calculated as total correct per level and overall. For each level, accuracy is calculated out of the full set of five possible items. While response time data is collected, it is not reported in this paper given the limited pool of data at higher levels of difficulty. The WMPT has previously been demonstrated to have good reliability (α = 0.84–0.85) and validity, and has been associated with achievement in numeracy, spelling, writing, and reading in Australian and Thai school children [69,70,72,89]. However, the validity of the measure has not been re-assessed after the implementation of the discontinue rule.

#### 2.3.5. Procedure

Parents completed the online survey either prior to or during their child’s face-to-face testing session on a personal electronic device which included the demographic questions and a measure assessing their child’s anxiety. For child participants, the order of administration of the WMPT, WIAT-III and another measure not included in this paper was counterbalanced using a rotating sequence across participants that included all 6 possible administration orders. Given the number of orders and sample size, administration order could not be meaningfully assessed as a source of variation. Across participants, 18 children completed the WMPT first, 15 completed the WIAT-III first, and 14 completed another measure prior to either the WMPT or WIAT-III.

The parent survey took approximately 60 min to complete, while testing time for children was around 90 min, including breaks between each assessment. To account for the effects of fatigue, children were encouraged to take breaks as often as needed to maintain engagement in the task. Parents were invited to remain with their child for the duration of the testing session, and children were not discouraged from fidgeting, stimming, or using self-regulation strategies throughout the testing session. Although not systematically recorded, observational impressions indicate that most parents chose to remain in the room during testing, and parent presence did not appear to differ by child neurotype, age, or anxiety level. All assessments were conducted by trained psychology researchers in a quiet testing room, following standard administration procedures for each measure.

The WMPT was completed on a laptop computer, using either the trackpad or mouse depending on child preference. Written instructions were presented at the beginning of each level and were verbally read to each child by researchers to ensure understanding. For the Memorise, 1-Swap and 2-Swap conditions, children completed two practice trials with feedback before commencing the test trials.

The WIAT-III subtests were administered using Q-interactive (Pearson Clinical Assessment, Sydney, Australia, versions 7.0 to 15.0) on an iPad, however the Numerical Operations subtest also required children to answer mathematical problems using a paper and pencil. Children completed the subtests in the same order: Reading Comprehension, Word Reading, Numerical Operations, then Mathematical Problem Solving. Specific reverse order administration and discontinue rules were followed for each subtest according to the WIAT-III manual. Scoring was completed automatically by Q-Interactive software.

#### 2.3.6. Data Analysis

All data analyses were conducted using JASP (version 0.19.3). Descriptive statistics were calculated for demographic characteristics, ASC-ASD total scores and subscales, WMPT accuracy, and the WIAT-III subtests. For the study measures, descriptive statistics are reported for the whole sample, as well as the neurodivergent and neurotypical subgroups. All analyses used listwise deletion. One participant had a single missing item from the ASC-ASD’s Uncertainty subscale. Consistent with the measure’s guidelines, this item was pro-rated using the mean of the completed items within that subscale, resulting in complete cases for all inferential analyses.

The primary preliminary analyses were specified a priori based on the study aims and hypotheses. These included group comparisons on the ASC-ASD, WMPT, and WIAT-III using independent samples *t*-tests (or Mann–Whitney U tests where appropriate), and repeated-measures ANOVAs to assess for interaction effects across WMPT levels. To directly examine the hypothesised relationships between anxiety, working memory, and academic achievement, correlations between the study measures were conducted for the whole sample, and linear regression models were run for each academic domain to assess whether anxiety and working memory accuracy predicted academic performance.

Secondary exploratory analyses included subgroup correlations for neurodivergent and neurotypical children. These analyses were considered exploratory due to the limited sample sizes, particularly for the neurodivergent group, and the potential imprecision of estimates.

The original analysis plan included subgroup regression models and the inclusion of covariates (e.g., age), as well as the reporting of analyses for the relationships between demographic and study variables. However, given the modest sample sizes and preliminary nature of the study, no additional covariates were included in the final regression models, and individual regression models for each neurotype were not included in this paper. These demographic analyses and the initial regression models which included child age as a covariate are provided in the Supplementary Material.

## 3. Results

### 3.1. Demographic Characteristics

As shown in Table 1, most reporting parents were women (85.11%), and all respondents were the biological parent of the study child. As shown in Table 2, approximately one third (*n* = 18; 38.30%) of children were reported to be or identified by their parent as being neurodivergent. Of these children, most were both autistic and ADHD (23.40% of total sample and 61.11% of the neurodivergent group).

All students attended school full-time and were enrolled across Grades 2–7. Most (93.62%) were enrolled in standard classes within mainstream schools. Of the remaining three students, one attended an autism-specific school (2.13%) and the other two (4.26%) were in support units within a mainstream school.

### 3.2. Anxiety

The mean scores for the ASC-ASD are presented in Table 3. Neurodivergent children scored higher than neurotypical children across all sub-domains and the total score, indicating greater overall and domain-specific anxiety.

Independent samples *t*-tests were used to compare group differences for the total score, but not the subscales. Mean differences were calculated as neurotypical means minus neurodivergent means. As all but the Performance Anxiety subscale violated normality assumptions and showed substantial skew, Mann–Whitney U tests were used for subscale comparisons. Statistically significant differences were observed for the ASC-ASD total score (*t*(45) = −2.20, *p* = 0.03, *MD* = −7.99, 95% CI [−15.30, −0.69], Hedges’ *g* = −0.65) and Uncertainty subscale (U = 137.50, *p* = 0.007, *r_rb_* = −0.47) for neurodivergent versus neurotypical students.

Children with reported diagnosed anxiety disorders also scored higher on the ASC-ASD total score and each subscale than those without such diagnoses. Significant differences were observed for the total score (*t*(45) = −3.684, *p* < 0.001, *MD* = −15.240, 95% CI [−23.571, −6.909], Hedges’ *g* = −1.343), and for all subscales (U values ranging 66.00 to 90.50, all *p* < 0.05).

As shown in Table 4, 25 (53.19%) children overall scored at or above the cut-point of 20 on the ASC-ASD, indicating elevated anxiety. For neurodivergent children, 13 (72.22%) met this threshold compared to 12 (41.38%) of the neurotypical children. Due to the small number of neurodivergent children who did not meet the cut-point, Fisher’s exact test was used to examine the association between neurotype and anxiety status. The result indicated higher odds of an elevated anxiety score amongst neurodivergent children, although the result was not statistically significant (OR = 3.58, 95% CI [0.89, 16.49], *p =* 0.07).

As shown in Table 5, the majority of children (88.89%) with a reported anxiety disorder exceeded the ASC-ASD’s cut point of ≥20. Fisher’s exact test indicated the association between having a reported anxiety disorder and meeting the ASC-ASD threshold for significant anxiety was significant (OR = 9.47, 95% CI [1.09, 457.75], *p* = 0.025).

### 3.3. Academic Achievement

The mean scores for the WIAT-III subtests assessing reading and mathematical achievement are presented in Table 6. Neurotypical children scored slightly higher across all assessed academic domains compared to neurodivergent children, with the exception of Numerical Operations. Independent samples *t*-tests were used to compare differences in academic achievement between groups, finding no significant differences: Word Reading (*t*(45) = 0.89, *p* = 0.38, *MD* = 4.36, 95% CI [−5.47, 14.19], Hedges’ *g* = 0.26); Reading Comprehension (*t*(45) = 1.64, *p* = 0.11, *MD* = 6.98, 95% CI [−1.60, 15.56], Hedges’ *g* = 0.48); Numerical Operations (*t*(45) = −0.12, *p* = 0.91, *MD* = −0.52, 95% CI [−9.46, 8.43], Hedges’ *g* = −0.03); and Math Problem Solving (*t*(45) = 0.83, *p* = 0.41, *MD* = 3.68, 95% CI [−5.21, 12.58], Hedges’ *g* = 0.25).

Further exploratory subgroup analyses indicated that there was a significant difference in performance on the Reading Comprehension subtest for autistic (n = 14) versus allistic children (i.e., children who were not autistic; n = 33; *M* = 92.07 versus *M* = 103.49, respectively), with a large effect size (*t*(45) = 2.63, *p* = 0.012, *MD* = 11.41, 95% CI [2.67, 20.16], Hedges’ *g* = 0.82). This significant difference also persisted when comparing AuDHD (n = 11; *M* = 90.46) students with neurotypical students (n = 29; *M* = 102.76; *t*(38) = 2.45, *p* = 0.02, *MD* = 12.30, 95% CI [2.08, 22.53], Hedges’ *g* = 0.85).

### 3.4. Working Memory

Mean accuracy for the WMPT for the whole sample was 62.3% (SD = 21.5%, range = 12–100%). Neurotypical children (*M* = 63.7%, *SD* = 21.2%) tended to have slightly higher overall accuracy, indicating marginally better working memory performance, compared to neurodivergent children (*M* = 60.0%, *SD* = 22.4%). Table 7 presents the number of children who reached each WMPT level separated by neurotype.

A repeated measures ANOVA was conducted to examine WMPT performance across the five difficulty levels for neurodivergent and neurotypical children. As Mauchly’s test indicated the assumption of sphericity was violated, Greenhouse–Geisser corrections were applied. There was a significant main effect for difficulty, indicating that accuracy decreased as working memory load increased, *F*(2.87, 128.95) = 82.40, *p* < 0.001, partial η^2^ = 0.647. Neither the main effect of group, *F*(1, 45) = 0.33, *p* = 0.57, partial η^2^ = 0.01, nor the interaction between difficulty and group, *F*(2.87, 128.95) = 0.240, *p* = 0.86, partial η^2^ = 0.01, were statistically significant. As shown in Figure 1, both neurodivergent and neurotypical groups showed a broadly comparable pattern of declining accuracy as task difficult increased.

### 3.5. Relationships Between Anxiety, Working Memory and Academic Achievement

The following correlational analyses represent the planned primary analyses examining the relationships between child anxiety, working memory, and academic achievement. The primary analyses to assess the hypotheses include the associations between the ASC-ASD total score, WMPT and academic outcomes. Associations between the ASC-ASD subscales and WMPT and academic measures, as well as correlations for the neurodivergent and neurotypical subgroups are exploratory. For exploratory correlations, Benjamini–Hochberg false discovery rate (FDR) corrections were applied (see Supplementary Material Tables S1–S3). Given that some of the ASC-ASD subscales were not normally distributed, Spearman’s rho was used for all correlational analyses to maintain consistency across variables. Confidence intervals (95%) were estimated using 1000 bootstrap replicates.

As shown in Table 8, for the whole sample, no statistically significant associations were observed between the ASC-ASD scores and the WMPT (ρ = 0.03, *p* = 0.86; 95% bootstrap CI [−0.25, 0.31]) or the WIAT-III Word Reading (ρ *=* 0.09, *p* = 0.55; 95% bootstrap CI [−0.21, 0.38]), Reading Comprehension (ρ *=* −0.15, *p* = 0.31; 95% bootstrap CI [−0.42, 0.17]), Numerical Operations (ρ *=* 0.12, *p* = 0.42; 95% bootstrap CI [−0.23, 0.45]) and Mathematical Problem Solving (ρ *=* 0.13, *p* = 0.38; 95% bootstrap CI [−0.15, 0.43]) subtests. Working memory performance, as measured by WMPT accuracy, was significantly and positively associated with scores on the Reading Comprehension (ρ *=* 0.41, *p* = 0.005; 95% bootstrap CI [0.09, 0.65]), Numerical Operations (ρ *=* 0.34, *p* = 0.021; 95% bootstrap CI [0.04, 0.59]) and Mathematical Problem Solving (ρ *=* 0.53, *p* < 0.001; 95% bootstrap CI [0.30, 0.71]) subtests, with each of these relationships being moderate to moderate-to-strong in strength. No significant association was detected between working memory and Word Reading (ρ *=* 0.24, *p* = 0.10; 95% bootstrap CI [−0.07, 0.51]).

Exploratory correlations between the study measures for neurodivergent children are presented in Table 9. Anxiety, as measured by the ASC-ASD total score, was not significantly associated with any of the achievement measures. Although some correlations appeared moderate in strength (e.g., Word Reading and Performance Anxiety; ρ *=* 0.48, *p* = 0.045; 95% bootstrap CI [−0.08, 0.82]), none reached statistical significance once FDR corrections were applied.

Working memory was positively related to the Separation Anxiety subscale (ρ *=* 0.48, *p* = 0.043; 95% bootstrap CI [0.003, 0.83]), and performance in Reading Comprehension (ρ *=* 0.64, *p* = 0.004; 95% bootstrap CI [0.25, 0.88]) and Maths Problem Solving (ρ *=* 0.64, *p* = 0.004; 95% bootstrap CI [0.23, 0.88]). However, after corrections, none of these correlations remained significant.

The exploratory correlations between the study measures for neurotypical children are shown in Table 10. Anxiety was not significantly associated with working memory, or any of the academic achievement scores. Notably, the correlations between the ASC-ASD total score and both the WMPT accuracy and Word Reading were negative for neurotypical children, in contrast to the positive associations observed in neurodivergent children. Working memory accuracy was moderately associated with Math Problem Solving (ρ *=* 0.49, *p* = 0.007; 95% bootstrap CI [0.17, 0.70]), but was not associated with any other achievement subtest. After FDR corrections, this correlation was not significant.

### 3.6. Anxiety and Working Memory as Predictors of Academic Achievement

A series of linear regressions were conducted to examine whether the ASC-ASD and WMPT predicted academic achievement in reading and mathematics.

The models for the whole sample are shown in Table 11. The models for Reading Comprehension, Numerical Operations, and Math Problem Solving were all significant, with the adjusted R^2^ values indicating that each model explained 24.9%, 14.9% and 27.4% of the variance in these achievement measures, respectively. Working memory was a significant predictor for all three of these models. Conversely, anxiety was not a significant predictor for achievement in any assessed domain.

## 4. Discussion

The aim of the present research was to conduct a preliminary investigation into the quantitative relationships between anxiety, working memory, and academic achievement in reading and mathematics in a mixed sample of neurodivergent and neurotypical children. Contrary to predictions, we did not detect statistically reliable associations between anxiety, as measured by the ASC-ASD, and academic achievement for the whole sample or for neurotypical students. For neurodivergent students, several uncorrected correlations were moderate in strength; however, most did not demonstrate statistically detectable effects, and none remained significant once false-discovery rate (FDR) corrections were applied. Given this, these patterns should be interpreted as descriptive and in need of further examination in larger, better powered samples.

Further contrary to expectations, anxiety was not negatively associated with working memory, as measured by the WMPT accuracy, for the whole sample. For neurotypical students, the relationships between working memory accuracy and anxiety did not reach significance although they were in the anticipated negative direction. For neurodivergent students, uncorrected correlations were positive in direction; however, the association between working memory and Separation Anxiety did not remain significant after FDR correction.

Lastly, largely in line with predictions, higher working memory accuracy was associated with increased achievement across Reading Comprehension, Numerical Operations and Math Problem Solving for the whole sample. Consistently, the regression models for these domains were significant, explaining between 15 to 27% of variance in achievement scores. However, no significant association was observed between working memory and Word Reading, and working memory was not a significant predictor in the relevant regression model. Although it must be acknowledged that the precision of these estimates is limited due to the small sample sizes, these patterns may tentatively indicate that, within this preliminary dataset, working memory may have a stronger association with academic performance for neurodivergent and neurotypical children than anxiety.

The lack of observed significant associations between anxiety and academic achievement in reading and numeracy is consistent with some previous research in neurotypical students, and may reflect the small, negative effect sizes observed in meta-analyses [33], which may have been largely unobservable in our limited sample. Other research has also suggested that test anxiety, rather than trait anxiety or anxiety disorder-based measures, is more likely to be related to performance on academic achievement tests, particularly when anxiety and achievement domains are in accordance (e.g., math anxiety is linked to performance on mathematical tasks; Refs. [90,91]). In addition, the positive nature of the association between anxiety and Word Reading for neurodivergent students was unexpected yet not unprecedented in neurotypical samples. For instance, one study by Alfonso and Lonigan [58] observed relationships between trait anxiety and passage comprehension in adolescents that were positive in direction, although these were not significant once other factors were accounted for. Moreover, this may reflect the idea that moderate levels of anxiety may be motivating for some children, or predispose them to engage more frequently in learning activities, e.g., refs. [22,92] to avoid anxiety relating to academic failure or embarrassing oneself in front of others.

For neurodivergent students in particular, the way that an academic test is administered may also influence results. For example, Lievore and Mammarella [46] reported that autistic students experienced more intense anxiety responses during a timed math task compared to their neurotypical peers due to the stress response elicited by this restriction, consequently displaying poorer performance on that task. As our tasks were untimed, often had parents present in the room, and allowed movement breaks, it is possible that aspects of the testing environment influenced children’s comfort and engagement. While this is one potential explanation, it must be noted that it cannot be definitively concluded that anxiety was reduced by these contextual factors in the present study, or that these factors enabled children to more accurately demonstrate their abilities. Accordingly, future research with larger samples should evaluate whether assessment contexts aligned with children’s sensory, emotional and physical needs meaningfully affect engagement and academic performance.

In terms of anxiety and working memory, the present findings align with individual studies that have not observed the detrimental impacts of anxiety on working memory performance in neurotypical children, but should be interpreted cautiously given that they diverge from meta-analytic evidence suggesting a reliable negative association [93]. In addition, while the uncorrected relationships between anxiety and working memory for neurodivergent children were positive in nature, none survived FDR correction and therefore cannot be interpreted as reliable effects. These preliminary patterns may reflect sample-specific variation, demographic characteristics or other contextual factors which were not statistically evaluated in this study. It is also possible that other findings related to the differing effects of anxiety depending on cognitive load could be relevant for understanding these patterns and examining these relationships in larger samples. For example, some research suggests that anxiety may be less impairing under higher cognitive load [94], although this was not tested in the current data. Given this, these potential mechanisms should be considered speculative, rather than established explanations. Other possibilities, including selection effects and measurement error, may also account for the observed results. Taken together, these preliminary patterns highlight the need for larger studies to examine cognitive–affective associations across neurotypes and testing contexts, rather than assuming uniform associations.

The overall significant positive associations observed between working memory and academic achievement in the domains of reading and numeracy are consistent with the large body of evidence demonstrating similar relationships, e.g., ref. [68,70,71,72]. In addition, the pattern of correlations across the whole sample suggests that while working memory demonstrated consistent associations with several achievement outcomes, anxiety was not reliably associated with academic achievement in this study. Further supporting this, all confidence intervals for anxiety-related correlations for the whole sample included zero, indicating that these estimates may be consistent with null associations.

However, two notable differences between the present research and the broader literature must be considered. First, we observed no significant differences in academic achievement or working memory accuracy between neurotypical and neurodivergent children. These findings differ from much of the literature that has reported working memory impairments or poorer academic performance in ADHD and autistic children compared to their neurotypical counterparts [95,96]. In addition, the finding that, for autistic and AuDHD children specifically, Reading Comprehension was the only domain where performance appeared to differ to comparison groups should be interpreted cautiously given the exploratory nature of the subgroup analyses, although it is broadly consistent with some previous findings [37,97]. Accordingly, larger studies are needed to clarify whether this pattern reflects true group differences.

One potential explanation for this difference is the requirement for understanding figurative or metaphorical language and abstract concepts to perform well on reading comprehension tasks, an area in which some autistic individuals may struggle [98,99]. As such figurative language is embedded within this subtest of the WIAT-III, some neurodivergent children within this study may have experienced difficulty with questions relating to abstract or metaphorical ideas, potentially contributing to the lower performance observed in this domain. In addition, this finding is somewhat similar to Bullen et al. [78], who observed an association with verbal working memory and both reading fluency and reading comprehension for a lower average achievement group of autistic students. Conversely, such relations were not observed for the high average achievement group in their study. Although we did not divide by achievement group, the range of scores in our neurodivergent sample indicates some children may have been classified as high achievers in this domain. The influence of co-occurring forms of neurodivergence should also be considered, as the majority of neurodivergent children in our sample were AuDHD children. In contrast, only two participants (8.7%) in Bullen et al.’s high achieving group had co-occurring ADHD. Future work should examine whether autistic students in general, but particularly those with co-occurring ADHD, may rely more heavily on working memory processes than their counterparts when engaging in reading comprehension tasks, as well as control for the potential influence of language abilities, including understanding of figurative language.

Secondly, differences were observed across task domains in line with task complexity. In particular, more complex tasks, such as Reading Comprehension and Math Problem Solving, which require individuals to actively hold and mentally manipulate relevant information extracted from text or combined verbal and visual stimuli, were more strongly associated with working memory accuracy than simpler tasks involving single word reading or basic arithmetic and mathematical functions. Although some research has reported stronger associations between working memory and mathematics rather than reading-based tasks [66,69,100], the similar strength of the associations with Reading Comprehension and Maths Problem Solving emerged as an interesting pattern. This result is broadly consistent with Davidson et al. [101], who reported that working memory was associated with reading comprehension in autistic and neurotypical children when considered alone. However, other studies have reported different patterns. For example, O’Leary et al. [77] found that working memory was not associated with reading comprehension in their sample of autistic students, but highlighted instead the roles of receptive grammar, non-verbal IQ and listening comprehension. In addition, other researchers have suggested that students may use different strategies to extract meaning from text dependent upon neurotype [102], and that working memory may help to support reading comprehension for students with language-related difficulties [103].

Cumulatively, these patterns and broader findings emphasise the need for future studies to examine the relationships between working memory and academic achievement across neurotypes and task domains. Although the subgroup findings were preliminary and based on small samples, the lower average performance on Reading Comprehension for autistic and AuDHD children specifically emerged as an interesting preliminary pattern. In addition, the effects of caregiver presence and movement breaks were not statistically evaluated. Given this, future research should also examine how assessment contexts and task demands influence children’s engagement with academic and cognitive tasks, and whether adjustments support more accurate demonstrations of ability across neurotypes.

Although this study included several strengths, such as the inclusion of a large proportion of children with co-occurring neurodivergent profiles and preliminary support for the feasibility of administering the WMPT to neurodivergent children, several important limitations must be acknowledged. First, the heterogenous composition of the neurodivergent group, combined with small subgroup sizes, limited the precision of estimates and restricts the extent to which neurotype-specific patterns can be interpreted. The use of a self-selected convenience sample also reduces generalisability, as families with particular experiences or interests may have been more likely to participate (e.g., those with more salient experiences with anxiety, or whose children were more proficient academically). In addition, neurotype and diagnoses were parent-reported. While this enabled a greater number of parent participants to participate in the online survey, it may have resulted in inaccurate reporting of neurodivergent identities.

Measurement-related limitations should also be considered. The ASC-ASD was developed specifically for autistic populations, and it is unclear whether the threshold score of ≥20 is similar across groups. Given this, the comparisons based on this measure should be interpreted cautiously, and future work should replicate the current findings using anxiety instruments with evidence of validity across multiple groups and informants. The WMPT discontinue rule resulted in missing response-time data at higher difficulty levels, and unadministered items were automatically scored as incorrect. This constrained our ability to examine response time patterns across neurotype and may have limited the interpretability of performance at higher difficulty levels. The effects of this newly implemented discontinue rule on the validity of the measure have also not been examined. Furthermore, the 90-min testing session may have introduced fatigue effects for some children, and the presence of caregivers during testing may have influenced engagement or anxiety levels. Additionally, anxiety was assessed via parent report. Although this assisted in limiting the testing burden placed on children, it may not have fully captured the children’s internal experiences.

Although preliminary analysis indicated that child age was associated with the ASC-ASD Uncertainty subscale, and that parent education and family income were related to academic outcomes (see Supplementary Material Section S3), these potential confounders were not feasible to include in the final regression models due to the modest sample sizes. Future research with larger samples should consider including these variables to more fully examine their potential confounding effects.

Finally, the cross-sectional design prevents conclusions about directionality or causality. Taken together, these limitations emphasise that the findings of this research should be considered preliminary and all interpretations are tentative in nature. Although some broad differences were observed in the patterns of associations between anxiety, working memory, and academic achievement in neurodivergent and neurotypical children, these exploratory findings require replication in larger, more diverse samples before firm conclusions can be drawn. These results should be viewed as a tentative pattern warranting further investigation rather than evidence of definitive group similarities or differences, as null findings may be due to limited statistical power to detect smaller effects rather than the absence of an underlying association.

Future adequately powered studies should examine potential mediatory pathways and consider whether cognitive–affective theories, such as Attentional Control Theory, apply to neurodivergent children. In addition, future research should consider broader academic outcomes beyond achievement which may be impacted by the elevated levels of anxiety observed in neurodivergent students compared to their neurotypical peers in other research, as well as the higher scores on the ASC-ASD seen in this study. As increased anxiety has been found to be associated with non-completion of high-school [33] and school can’t or attendance problems in neurotypical students [9,104], it should be investigated whether such relationships exist in neurodivergent students and contribute to the observed gaps in academic attainment, particularly in relation to tertiary qualifications, e.g., ref. [105]. Furthermore, the potential impacts of anxiety on other important factors for neurodivergent children, such as mental health and wellbeing, should also be considered.

In conclusion, this study provides a preliminary mapping of cognitive–affective associations across neurodivergent and neurotypical children, including a substantial subgroup with co-occurring neurodevelopmental profiles. Although limited by sample size, the exploratory patterns observed across neurotype highlight the need for further research to clarify the potential roles of cognitive and affective factors in the academic achievement of neurodivergent children, as well as examine how assessment contexts may influence how some neurodivergent children engage with and perform on cognitive and academic tasks. Given the central role of anxiety in child mental health and school functioning, these findings underscore the importance of considering how affective factors, assessment environments and cognitive demands interact for neurodivergent children, especially in contexts where clinicians and educators collaborate to support learning and wellbeing.

## Figures and Tables

**Figure 1:**
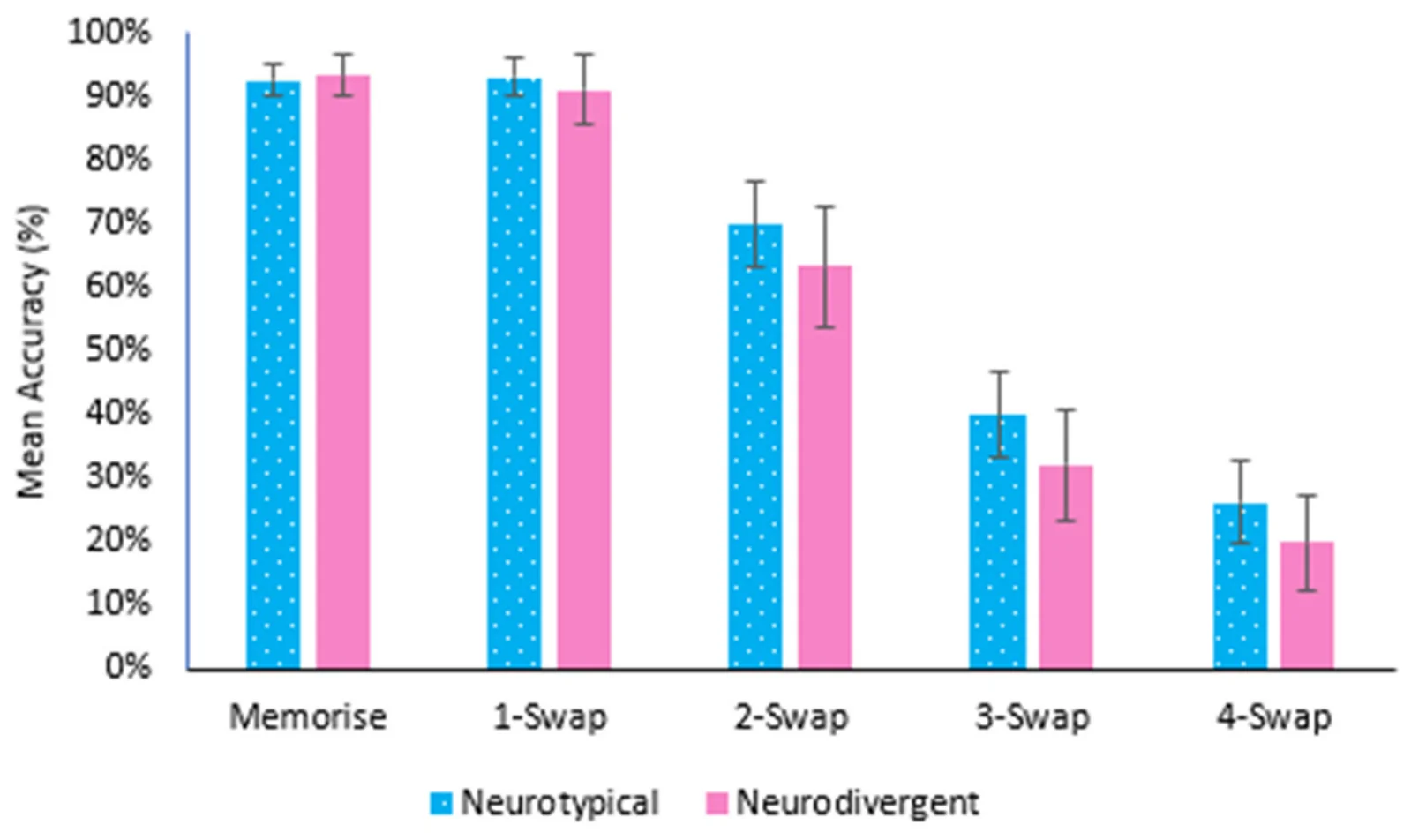
Mean accuracy for each level of difficulty for the Working Memory Power Test for neurotypical and neurodivergent children. Errors bars represent standard errors. Note: Discontinue rule scored unadministered items as 0.

**Table 1:** Parent demographic characteristics reported by parent (*n* = 47).

	*n*	%
Gender		
Woman	40	85.11%
Man	7	14.89%
Geographic location		
Metropolitan/Inner City	7	14.89%
Urban/Outer Suburbs	21	44.68%
Regional/Remote	19	40.43%
Relationship status		
Single	7	14.89%
Living together/married	36	76.60%
Separated/divorced	4	8.51%
Aboriginal and/or Torres Strait Islander		
Yes	4	8.51%
Highest education level		
Year 12 or lower	8	17.02%
TAFE Certificate	10	21.28%
Diploma	8	17.02%
Bachelor Degree	15	31.92%
Master’s Degree	6	12.77%
Employment		
Not currently employed ^a^	12	25.53%
Part time employed	20	42.55%
Full time employed	15	31.92%
Family income (AUD) ^b^		
$20,000–50,000	7	15.22%
$50,001–75,000	4	8.70%
$75,001–100,000	8	17.39%
$100,001–150,000	10	21.74%
>$150,001	17	36.96%

^a^ This includes parents who reported they were full-time parents, currently unemployed, or students. ^b^ One parent did not report their family income.

**Table 2:** Child demographic characteristics reported by parent (*n* = 47).

	*n*	%
Gender		
Girl	26	55.32%
Boy	20	42.55%
Non-binary	1	2.13%
Neurodivergence		
Autism only	3	6.38%
ADHD only	3	6.38%
Autism and ADHD	11	23.40%
Dyslexia and Auditory Processing Disorder	1	2.13%
Neurotypical	29	61.70%
Mental health/other conditions		
Anxiety disorder	9	19.15%
Depressive disorder	1	2.13%
Intellectual disability	1	2.13%
Access to teacher’s aide		
Yes	13	28.26%
No	33	71.74%
Did not respond	1	
School can’t frequency ^a^		
Never	17	36.17%
A few times each month	18	38.30%
Weekly	7	14.89%
A few days each week	3	6.38%
Daily	2	4.26%
School exclusion history		
Suspended	2	4.26%

^a^ “School can’t frequency” reflects parent responses to the questionnaire item asking how often their child refuses or attempts to avoid attending school.

**Table 3:** Means, standard deviations, and range of scores for the ASC-ASD, presented for the total sample, and neurodivergent and neurotypical participants.

	Total		Neurodivergent		Neurotypical	
	M (SD)	Range	M (SD)	Range	M (SD)	Range
Total Score *	22.23 (12.59)	3–50	27.17 (13.13)	3–45	19.17 (11.42)	4–50
Anxious Arousal	2.60 (3.12)	0–13	3.61 (3.56)	0–10	1.97 (2.69)	0–13
Performance Anxiety	6.89 (3.89)	0–14	7.22 (4.25)	0–14	6.69 (3.70)	1–14
Separation Anxiety	5.11 (4.06)	0–15	5.94 (3.80)	0–14	4.59 (4.20)	0–15
Uncertainty *	7.64 (5.26)	0–22	10.39 (6.04)	0–22	5.93 (3.93)	0–13

* Indicates a significant difference (*p* < 0.05).

**Table 4:** Contingency table of ASC-ASD threshold groups by neurotype.

	Below ASC-ASD Threshold	Meets ASC-ASD Threshold
Neurodivergent	5	13
Neurotypical	17	12

**Table 5:** Contingency table of ASC-ASD threshold groups by reported anxiety disorder.

	Below ASC-ASD Threshold	Meets ASC-ASD Threshold
No anxiety disorder	21	17
Anxiety disorder	1	8

**Table 6:** Means, standard deviations, and range of scores for the WIAT-III Word Reading, Reading Comprehension, Numerical Operations, and Mathematical Problem Solving subtests, presented for the total sample, and neurodivergent and neurotypical participants.

	Total		Neurodivergent		Neurotypical	
	M (SD)	Range	M (SD)	Range	M (SD)	Range
Word Reading	109.75 (16.22)	73–148	107.06 (17.84)	73–133	111.41 (15.22)	80–148
Reading Comp.	100.08 (14.46)	48–142	95.78 (16.40)	48–120	102.76 (12.69)	78–142
Numerical Ops.	99.68 (14.64)	54–135	100.00 (17.42)	54–127	99.48 (12.95)	72–135
Math Prob. Solving	100.11 (14.67)	52–132	97.83 (17.06)	52–129	101.52 (13.09)	73–132

**Table 7:** Number of children who reached each level of the Working Memory Power Test, presented for neurodivergent and neurodivergent participants.

	Neurodivergent	Neurotypical
Memorise	18	29
1-Swap	18	29
2-Swap	17	28
3-Swap	14	24
4-Swap	8	15

**Table 8:** Correlations between the ASC-ASD total score and subscales, the WMPT, and the WIAT-III subtests for the whole sample (n = 47).

	1	2	3	4	5	6	7	8	9
1. ASC-ASD Total									
2. AA Subscale	0.71 ***								
3. PA Subscale	0.78 ***	0.56 ***							
4. SA Subscale	0.76 ***	0.36 *	0.46 **						
5. Uncertainty Subscale	0.84 ***	0.49 ***	0.52 ***	0.52 ***					
6. Working Memory	0.03	−0.18	0.15	0.04	0.01				
7. Word Reading	0.09	−0.10	0.04	0.14	0.09	0.24			
8. Reading Comp.	−0.15	−0.10	−0.05	−0.21	−0.18	0.41 **	0.17		
9. Numerical Ops.	0.12	−0.06	0.09	0.08	0.14	0.34 *	0.44 **	0.33 *	
10. Math Prob. Solving	0.13	0.05	0.17	0.08	−0.01	0.53 ***	0.34 *	0.53 ***	0.65 ***

* *p* < 0.05, ** *p* < 0.01, *** *p* < 0.001. Note: ASC-ASD = Anxiety Scale for Children with Autism Spectrum Disorder; AA = Anxious Arousal; PA = Performance Anxiety; SA = Separation Anxiety.

**Table 9:** Correlations between the ASC-ASD total score and subscales, the WMPT, and the WIAT-III subtests for neurodivergent children (n = 18).

	1	2	3	4	5	6	7	8	9
1. ASC-ASD Total									
2. AA Subscale	0.60 **								
3. PA Subscale	0.75 ***	0.44							
4. SA Subscale	0.83 ***	0.58 *	0.66 **						
5. Uncertainty Subscale	0.78 ***	0.25	0.40	0.43					
6. Working Memory	0.46	−0.01	0.43	0.48	0.22				
7. Word Reading	0.46	0.12	0.48	0.20	0.41	0.34			
8. Reading Comp.	−0.06	−0.31	0.10	−0.13	−0.10	0.64	0.23		
9. Numerical Ops.	0.25	0.00	0.30	0.12	0.12	0.35	0.61 **	0.41	
10. Math Prob. Solving	0.39	0.15	0.27	0.33	0.18	0.64	0.54 *	0.48 *	0.76 ***

* *p* < 0.05, ** *p* < 0.01, *** *p* < 0.001. Note: Significant markers have been removed for correlations that did not remain significant after FDR correction. Correlations between the ASC-ASD subscales and between the WIAT-III subtests are descriptive and were not included in the FDR corrections. ASC-ASD = Anxiety Scale for Children with Autism Spectrum Disorder; AA = Anxious Arousal; PA = Performance Anxiety; SA = Separation Anxiety.

**Table 10:** Correlations between the ASC-ASD total score and subscales, the WMPT, and the WIAT-III subtests for neurotypical children (n = 29).

	1	2	3	4	5	6	7	8	9
1. ASC-ASD Total									
2. AA Subscale	0.76 ***								
3. PA Subscale	0.86 ***	0.72 ***							
4. SA Subscale	0.58 ***	0.16	0.30						
5. Uncertainty Subscale	0.86 ***	0.59 ***	0.66 ***	0.38 *					
6. Working Memory	−0.18	−0.26	−0.04	−0.14	−0.07				
7. Word Reading	−0.18	−0.28	−0.30	0.10	−0.08	0.18			
8. Reading Comp.	−0.11	0.15	−0.11	−0.22	−0.07	0.30	0.11		
9. Numerical Ops.	0.04	−0.11	0.06	0.03	0.14	0.26	0.28	0.26	
10. Math Prob. Solving	0.07	0.07	0.14	0.03	−0.04	0.49	0.16	0.62 ***	0.56 **

* *p* < 0.05, ** *p* < 0.01, *** *p* < 0.001. Note: Significant markers have been removed for correlations that did not remain significant after FDR correction. Correlations between the ASC-ASD subscales and between the WIAT-III subtests are descriptive and were not included in the FDR corrections. ASC-ASD = Anxiety Scale for Children with Autism Spectrum Disorder; AA = Anxious Arousal; PA = Performance Anxiety; SA = Separation Anxiety.

**Table 11:** Results of the regression model using anxiety and working memory scores to predict academic achievement for the whole sample.

Domain	Predictors	B	*SE*	95% CI	*R*	*R* ^2^	*R* ^2^ *_adj_*	*F*	*β*	*t*	*p*
Word Reading					0.278	0.077	0.035	1.84			0.171
	Intercept	94.82	8.25	78.19, 111.44						11.50	<0.001
	ASC-ASD	0.12	0.19	−0.25, 0.50					0.10	0.66	0.513
	WMPT	19.57	10.94	−2.47, 41.61					0.26	1.79	0.080
Reading Comp.					0.531	0.282	0.249	8.62			<0.001
	Intercept	83.14	6.49	70.07, 96.22						12.82	<0.001
	ASC-ASD	−0.19	0.15	−0.49, 0.10					−0.17	−1.31	0.196
	WMPT	34.07	8.60	16.74, 51.41					0.51	3.96	<0.001
Num. Op.					0.431	0.186	0.149	5.03			0.011
	Intercept	78.91	6.99	64.82, 93.00						11.29	<0.001
	ASC-ASD	0.16	0.16	−0.16, 0.48					0.14	0.99	0.328
	WMPT	27.76	9.27	9.08, 46.44					0.41	3.00	0.004
Math Prob. Sol.					0.553	0.306	0.274	9.69			<0.001
	Intercept	73.78	6.47	60.74, 86.82							<0.001
	ASC-ASD	0.17	0.15	−0.13, 0.46					0.15	1.15	0.256
	WMPT	36.25	8.58	18.97, 53.53					0.53	4.23	<0.001

Note: All regression models n = 47; df = 2, 44. WMPT scores entered as a percentage (0–100%).

## Data Availability

The de-identified dataset supporting the conclusions of this article will be made available by the authors upon reasonable request. Any data sharing will occur in accordance with institutional ethics approval and privacy requirements.

## Supplementary Materials

The following supporting information can be downloaded at: https://www.mdpi.com/article/10.3390/children13101440/s1.
