# Supplementary material for: Anxiety, Working Memory, and Academic Achievement in Neurodivergent and Neurotypical Children: A Preliminary Study
Source: Children (Basel). 2026 Oct 21;13(10):1440. doi: 10.3390/children13101440 (PMC13703206; doi:10.3390/children13101440)
Supplement: Supplementary file 1 [file children-13-01440-s001.zip › children-4589021-supplementary.pdf]

Supplementary Material

Section S1. Benjamini-Hochberg False Detection Rate (FDR) Tables.

Table S1. Benjamini-Hochberg adjusted p values for exploratory correlations for the whole sample (n = 47).

Variables	Original P value	Critical Value	Benjamini-Hochberg Adjusted P value	Significant after FDR (0.05)
SA Subscale + Reading Comp.	0.158	0.0025000000000000005	0.8666666666666667	No
Uncertainty + Reading Comp.	0.231	0.005000000000000001	0.8666666666666667	No
AA Subscale + Working Memory	0.234	0.0075	0.8666666666666667	No
PA Subscale + Math Prob. Solv.	0.256	0.010000000000000002	0.8666666666666667	No
PA Subscale + Working Memory	0.313	0.0125	0.8666666666666667	No
Uncertainty + Numerical Ops.	0.349	0.015	0.8666666666666667	No
SA Subscale + Word Reading	0.365	0.017499999999999998	0.8666666666666667	No
AA Subscale + Reading Comp.	0.493	0.020000000000000004	0.8666666666666667	No
AA Subscale + Word Reading	0.502	0.022500000000000003	0.8666666666666667	No
Uncertainty + Word Reading	0.528	0.025	0.8666666666666667	No
PA Subscale + Numerical Ops.	0.548	0.027500000000000004	0.8666666666666667	No
SA Subscale + Math Prob. Solv.	0.579	0.03	0.8666666666666667	No

SA Subscale + Numerical Ops.	0.607	0.0325	0.8666666666666667	No
AA Subscale + Numerical Ops.	0.709	0.034999999999999996	0.8666666666666667	No
PA Subscale + Reading Comp.	0.728	0.037500000000000006	0.8666666666666667	No
AA Subscale + Math Prob. Solv.	0.735	0.04000000000000001	0.8666666666666667	No
SA Subscale + Working Memory	0.767	0.0425	0.8666666666666667	No
PA Subscale + Word Reading	0.78	0.045000000000000005	0.8666666666666667	No
Uncertainty + Math Prob. Solv.	0.931	0.0475	0.962	No
Uncertainty + Working Memory	0.962	0.05	0.962	No

Note: ASC-ASD = Anxiety Scale for Children with Autism Spectrum Disorder; AA = Anxious Arousal; PA = Performance Anxiety; SA = Separation Anxiety

Table S2. Benjamini-Hochberg adjusted p values for exploratory correlations for the neurodivergent sample (n = 18).

Variables	Original P value	Critical Value	Benjamini-Hochberg Adjusted P value	Significant after FDR (0.05)
Working Memory + Math Prob. Solv.	0.004	0.0017241379310344827	0.058	No
Working Memory + Reading Comp.	0.004	0.0034482758620689655	0.058	No
SA Subscale + Working Memory	0.043	0.005172413793103448	0.27549999999999997	No
PA Subscale + Word Reading	0.045	0.006896551724137931	0.27549999999999997	No
ASC-ASD Total + Working Memory	0.055	0.008620689655172415	0.27549999999999997	No

ASC-ASD Total + Word Reading	0.057	0.010344827586206896	0.27549999999999997	No
PA Subscale + Working Memory	0.073	0.012068965517241381	0.30242857142857144	No
Uncertainty + Word Reading	0.095	0.013793103448275862	0.344375	No
ASC-ASD Total + Math Prob. Solv.	0.114	0.015517241379310346	0.36733333333333335	No
Working Memory + Numerical Ops.	0.153	0.01724137931034483	0.43258333333333326	No
Working Memory + Word Reading	0.167	0.01896551724137931	0.43258333333333326	No
SA Subscale + Math. Prob. Solv.	0.179	0.020689655172413793	0.43258333333333326	No
AA Subscale + Reading Comp.	0.204	0.02241379310344828	0.45507692307692305	No
PA Subscale + Numerical Ops.	0.22	0.024137931034482762	0.45571428571428574	No
PA Subscale + Math Prob. Solv.	0.281	0.025862068965517244	0.5432666666666667	No
ASC-ASD Total + Numerical Ops.	0.327	0.027586206896551724	0.5926875	No
Uncertainty + Working Memory	0.382	0.029310344827586206	0.6516470588235294	No
SA Subscale + Word Reading	0.439	0.031034482758620693	0.7072777777777778	No
Uncertainty + Math Prob. Solv.	0.471	0.032758620689655175	0.7188947368421053	No
AA Subscale + Math Prob. Solv.	0.556	0.03448275862068966	0.7733333333333333	No
SA Subscale + Reading Comp.	0.597	0.03620689655172414	0.7733333333333333	No

AA Subscale + Word Reading	0.623	0.03793103448275862	0.7733333333333333	No
Uncertainty + Numerical Ops.	0.623	0.039655172413793106	0.7733333333333333	No
SA Subscale + Numerical Ops.	0.64	0.041379310344827586	0.7733333333333333	No
PA Subscale + Reading Comp.	0.68	0.04310344827586207	0.7774230769230769	No
Uncertainty + Reading Comp.	0.697	0.04482758620689656	0.7774230769230769	No
ASC-ASD Total + Reading Comp.	0.802	0.04655172413793104	0.8614074074074075	No
AA Subscale + Working Memory	0.957	0.048275862068965524	0.987	No
AA Subscale + Numerical Ops.	0.987	0.05	0.987	No

Note: ASC-ASD = Anxiety Scale for Children with Autism Spectrum Disorder; AA = Anxious Arousal; PA = Performance Anxiety; SA = Separation Anxiety

Table S3. Benjamini-Hochberg adjusted p values for exploratory correlations for the neurotypical sample (n = 18).

Variables	Original P value	Critical Value	Benjamini-Hochberg Adjusted P value	Significant after FDR (0.05)
Working Memory + Math Prob. Solv.	0.007	0.0017241379310344827	0.203	No
PA Subscale + Word Reading	0.114	0.0034482758620689655	0.8409999999999999	No
Working Memory + Reading Comp.	0.12	0.005172413793103448	0.8409999999999999	No
AA Subscale + Word Reading	0.138	0.006896551724137931	0.8409999999999999	No
Working Memory + Numerical Ops.	0.167	0.008620689655172415	0.8409999999999999	No

AA Subscale + Working Memory	0.174	0.010344827586206896	0.8409999999999999	No
SA Subscale + Reading Comp.	0.253	0.012068965517241381	0.8905416666666666	No
ASC-ASD Total + Word Reading	0.357	0.013793103448275862	0.8905416666666666	No
Working Memory + Word Reading	0.361	0.015517241379310346	0.8905416666666666	No
ASC-ASD Total + Working Memory	0.364	0.01724137931034483	0.8905416666666666	No
AA Subscale + Reading Comp.	0.444	0.01896551724137931	0.8905416666666666	No
SA Subscale + Working Memory	0.464	0.020689655172413793	0.8905416666666666	No
Uncertainty + Numerical Ops.	0.468	0.02241379310344828	0.8905416666666666	No
PA Subscale + Math Prob. Solv.	0.472	0.024137931034482762	0.8905416666666666	No
PA Subscale + Reading Comp.	0.568	0.025862068965517244	0.8905416666666666	No
ASC-ASD Total + Reading Comp.	0.57	0.027586206896551724	0.8905416666666666	No
AA Subscale + Numerical Ops.	0.589	0.029310344827586206	0.8905416666666666	No
SA Subscale + Word Reading	0.61	0.031034482758620693	0.8905416666666666	No
Uncertainty + Word Reading	0.699	0.032758620689655175	0.8905416666666666	No
ASC-ASD Total + Math Prob. Solv.	0.718	0.03448275862068966	0.8905416666666666	No
Uncertainty + Reading Comp.	0.722	0.03620689655172414	0.8905416666666666	No

AA Subscale + Math Prob. Solv.	0.731	0.03793103448275862	0.8905416666666666	No
PA Subscale + Numerical Ops.	0.734	0.039655172413793106	0.8905416666666666	No
Uncertainty + Working Memory	0.737	0.041379310344827586	0.8905416666666666	No
Uncertainty + Math Prob. Solv.	0.825	0.04310344827586207	0.898	No
PA Subscale + Working Memory	0.835	0.04482758620689656	0.898	No
ASC-ASD Total + Numerical Ops.	0.839	0.04655172413793104	0.898	No
SA Subscale + Numerical Ops.	0.895	0.048275862068965524	0.898	No
SA Subscale + Math Prob. Solv.	0.898	0.05	0.898	No

Note: ASC-ASD = Anxiety Scale for Children with Autism Spectrum Disorder; AA = Anxious Arousal; PA = Performance Anxiety; SA = Separation Anxiety

Section S2. Age Included and Neurotype-Specific Regression Models

Table S4. Results of the regression model using child age, anxiety and working memory scores to predict academic achievement for the whole sample.

Domain	Predictors	B	*SE*	95% CI	*R*	R^2	R^2_{adj}	*F*	*β*	*t*	*p*
Word Reading					0.36	0.13	0.07	2.19			0.103
	Intercept	114.69	14.45	85.55, 143.83						7.94	<0.001
	Age	-2.36	1.42	-5.24, 0.51					-0.24	-1.66	0.104
	ASC-ASD	0.16	0.18	-0.21, 0.53					0.13	0.87	0.388
	WMPT	23.61	11.00	1.43, 45.78					0.31	2.15	0.037
Reading Comp.					0.56	0.31	0.27	6.55			<0.001
	Intercept	96.61	11.46	73.50, 119.71						8.43	<0.001
	Age	-1.60	1.13	-3.88, 0.68					-0.19	-1.42	0.163
	ASC-ASD	-0.17	0.15	-0.46, 0.13					-0.15	-1.14	0.260
	WMPT	36.81	8.72	19.23, 54.39					0.55	4.22	<0.001
Num. Op.					0.46	0.21	0.15	3.77			0.017
	Intercept	90.23	12.46	65.11, 115.35						7.24	<0.001
	Age	-1.35	1.23	-3.82, 1.13					-0.15	-1.10	0.279
	ASC-ASD	0.18	0.16	-0.14, 0.50					0.15	1.12	0.269
	WMPT	30.06	9.48	10.94, 49.18					0.44	3.17	0.003
Math Prob. Sol.					0.58	0.34	0.30	7.43			<0.001
	Intercept	88.18	11.38	65.23, 111.14						7.75	<0.001
	Age	-1.71	1.12	-3.98, .55					-0.20	-1.53	0.134
	ASC-ASD	0.20	0.15	-0.10, 0.49					0.17	1.35	0.185
	WMPT	39.18	8.66	21.71, 56.65					0.57	4.52	<0.001

Note: All regression models n = 47; df = 3, 43. WMPT scores entered as a percentage (0-100%).

Table S5. Results of the regression model using child age, anxiety and working memory scores to predict academic achievement for the neurodivergent children.

Domain	Predictors	B	*SE*	95% CI	*R*	R^2	R^2_{adj}	*F*	*β*	*t*	*p*
Word Reading					0.51	0.26	0.10	1.60			0.235
	Intercept	88.55	26.01	32.77, 144.33						3.41	0.004

	Age	-0.83	2.66	-6.52, 4.87					-0.08	-0.31	0.760
	ASC-ASD	0.43	0.35	-0.34, 1.19					0.31	1.20	0.251
	WMPT	25.04	19.85	-17.53, 67.61					0.32	1.26	0.228
Reading Comp.					0.77	0.59	0.50	6.66			0.005
	Intercept	86.06	17.77	47.95, 124.17						4.84	<0.001
	Age	-1.66	1.81	-5.55, 2.23					-0.17	-0.92	0.376
	ASC-ASD	-0.36	0.24	-0.88, 0.16					-0.29	-1.50	0.156
	WMPT	59.53	13.56	30.44, 88.61					0.81	4.39	<0.001
Num. Op.					0.67	0.45	0.33	3.81			0.035
	Intercept	119.09	21.83	72.26, 165.91						5.46	<0.001
	Age	-5.13	2.23	-9.91, -0.34					-0.48	-2.30	0.037
	ASC-ASD	0.42	0.30	-0.22, 1.06					0.32	1.41	0.181
	WMPT	32.26	16.66	-3.47, 67.99					0.42	1.94	0.073
Math Prob. Sol.					.72	.52	.42	5.08			0.014
	Intercept	104.40	19.93	61.65, 147.16						5.24	<0.001
	Age	-4.24	2.04	-8.60, 0.13					-0.41	-2.08	0.056
	ASC-ASD	0.34	0.27	-0.24, 0.93					0.26	1.26	0.229
	WMPT	42.24	15.21	9.61, 74.86					0.56	2.78	0.015

Note: All regression models n = 18; df = 3, 14. WMPT scores entered as a percentage (0-100%).

Table S6. Results of the regression model using child age, anxiety and working memory scores to predict academic achievement for neurotypical children.

**Domain**	**Predictors**	**B**	***SE***	**95% CI**	***R***	R^2	R^2_{adj}	***F***	β	***t***	***p***
Word Reading					0.40	0.16	0.06	1.55			0.226
	Intercept	135.13	17.66	98.76, 171.50						7.65	<0.001
	Age	-3.45	1.69	-6.94, 0.04					-0.39	-2.04	0.052
	ASC-ASD	-0.003	0.25	-0.52, 0.51					-0.002	-0.01	0.992
	WMPT	16.45	14.03	-12.44, 45.34					0.23	1.17	0.252
Reading Comp.					0.39	0.16	0.06	1.53			0.232
	Intercept	101.34	14.74	70.98, 131.69						6.88	<0.001

	Age	-1.21	1.41	-4.12, 1.70					-0.16	-0.86	0.401
	ASC-ASD	-0.07	0.21	-0.50, 0.36					-0.06	-0.33	0.748
	WMPT	23.05	11.71	-1.07, 47.16					0.39	1.97	0.060
Num. Op.					0.39	0.15	0.05	1.45			0.251
	Intercept	77.88	14.10	46.77, 108.99						5.16	<0.001
	Age	0.67	1.45	-2.32, 3.65					0.09	0.46	0.650
	ASC-ASD	0.06	0.21	-0.39, 0.50					0.05	0.26	0.798
	WMPT	21.89	12.00	-2.82, 46.61					0.36	1.83	0.080
Math Prob. Sol.					0.51	0.26	0.17	2.96			0.051
	Intercept	81.00	14.21	51.73, 110.27						5.70	<0.001
	Age	-0.45	1.36	-3.26, 2.36					-0.06	-0.33	0.744
	ASC-ASD	0.21	0.20	-0.20, 0.63					0.19	1.06	0.300
	WMPT	32.77	11.29	9.52, 56.02					0.53	2.90	0.008

Note: All regression models n = 29; df = 3, 25. WMPT scores entered as a percentage (0-100%).

Section S3. Exploratory Relationships between Demographic Characteristics and Study Measures

Pearson or Spearman's correlation analyses were used to examine the relationships between the study measures and parent and child demographic variables. For binary variables, independent samples *t*-tests were used. Of the examined parent demographic factors, only parent education level and income were related to the study measures. Specifically, parent education was positively associated with child scores on the Reading Comprehension ($\varrho = 0.381$, $p = 0.008$), Word Reading ($\varrho = 0.415$, $p = 0.004$) and Mathematical Problem Solving subtests ($\varrho = 0.327$, $p = 0.025$), indicating that children with more highly educated parents tended to have higher levels of achievement. Family income was also positively correlated with Mathematical Problem Solving ($\varrho = 0.356$, $p = 0.015$).

Regarding child demographic factors, child age and gender were not significantly related to any of the study measures with one exception. Child age was positively associated with the ASC-ASD's Uncertainty subscale, suggesting that older children tended to experience more anxiety related to uncertainty ($\varrho = .344$, $p = .018$). As there was only one non-binary participant, they were excluded from the gender analyses due to an insufficient sample size for accurate inferences.

Having a teacher's aide was related to lower reading, but not numeracy performance. Compared to children without access to a teacher's aide, children with an aide scored significantly lower in Reading Comprehension ($M = 102.91$, $SD = 12.17$ versus $M = 93.54$, $SD = 18.24$; $t(44) = -2.03$, $p = 0.048$) and Word Reading ($M = 113.06$, $SD = 14.12$ versus $M = 101.69$, $SD = 19.37$; $t(44) = -2.21$, $p = 0.033$).
